# Low Body Mass Index as a Significant Risk Factor for Hypoglycemia in Hospitalized Elderly Patients With Acute Pyelonephritis: A Retrospective Cohort Study

**DOI:** 10.7759/cureus.72682

**Published:** 2024-10-30

**Authors:** Satoru Sekiguchi, Ryuichi Ohta, Chiaki Sano

**Affiliations:** 1 Family Medicine, Shimane University Faculty of Medicine, Izumo, JPN; 2 Community Care, Unnan City Hospital, Unnan, JPN; 3 Community Medicine Management, Shimane University Faculty of Medicine, Izumo, JPN

**Keywords:** acute pyelonephritis, body mass index, elderly, general medicine, hospitalization, hypoglycemia, risk factors, rural

## Abstract

Introduction

Hypoglycemia is a significant clinical concern among hospitalized elderly patients, particularly those with acute illnesses such as pyelonephritis. It is associated with increased morbidity and mortality. Identifying specific risk factors for hypoglycemia in this vulnerable population is crucial to developing targeted interventions and improving patient outcomes. This study aimed to determine the risk factors associated with hypoglycemic events in elderly patients hospitalized with acute pyelonephritis.

Methods

This retrospective cohort study included 294 patients aged 65 years and older who were admitted to Unnan City Hospital with a diagnosis of acute pyelonephritis between January 2021 and April 2024. Data on demographic characteristics, comorbidities, and clinical outcomes were extracted from medical records. The primary outcome was the occurrence of hypoglycemia, defined as a blood glucose level below 70 mg/dL during hospitalization. Multivariate logistic regression analysis was performed to identify independent risk factors associated with hypoglycemic events.

Results

Hypoglycemia occurred in 146 (49.7%) of the 294 hospitalized patients. Lower body mass index (BMI) was significantly associated with an increased risk of hypoglycemia (OR = 0.84, 95% CI: 0.78-0.90, p < 0.00001). Age, sex, and comorbidities such as diabetes, heart failure, and chronic obstructive pulmonary disease did not significantly predict hypoglycemia risk. Hypoglycemic patients had a higher in-hospital mortality rate compared to non-hypoglycemic patients (46.6% vs. 31.1%, p = 0.008).

Conclusions

Lower BMI is an independent risk factor for hypoglycemia in elderly patients hospitalized with acute pyelonephritis. Early identification and tailored management of patients with low BMI could reduce the incidence of hypoglycemic events and improve clinical outcomes in this high-risk population.

## Introduction

Hypoglycemia is a common occurrence among hospitalized patients and is associated with poor prognosis, including increased mortality rates [1]. Older adults and those with comorbid conditions are particularly vulnerable to the adverse effects of hypoglycemia [2]. The susceptibility to hypoglycemia in elderly patients can be attributed to several factors, such as the increased prevalence of comorbidities requiring multiple medications, physiological changes associated with aging, and a progressive decline in overall health [3]. Previous studies have reported that elderly diabetic patients with a low body mass index (BMI) frequently experience hypoglycemia [4]. This condition has been linked to prolonged hospital stays and a decline in glycemic control after discharge [5].

Given these findings, it is essential to identify the risk factors associated with hypoglycemic events in hospitalized elderly patients to develop preventive measures and improve patient outcomes. However, there is little research focusing on hypoglycemia among older hospitalized patients without diabetes. As societies are aging, hospitalized patients’ age is becoming higher, and they have frailty and multimorbidity with polypharmacy [6,7]. Even though they do not have diabetes with medicines, hypoglycemic episodes can be triggered by acute diseases complicated by their chronic diseases [8].

We analyzed data from hospitalized patients at Unnan City Hospital to address this issue. Our study focused on urinary tract infection patients without diabetes because the infection is common and triggers sepsis, causing hypoglycemia among older patients. By understanding the factors that contribute to hypoglycemia during hospitalization, early intervention through the administration of intravenous fluids or other measures could prevent hypoglycemic episodes, thereby improving patient prognosis. This study aims to identify the specific risk factors contributing to hypoglycemia in hospitalized elderly patients. By analyzing patient data, including demographic information, comorbid conditions, medication use, and clinical outcomes, this research seeks to provide a comprehensive understanding of the determinants of hypoglycemia in elderly inpatients.

## Materials and methods

Study design

This retrospective cohort study investigated the factors associated with hypoglycemic events in elderly patients aged 65 years and older hospitalized with pyelonephritis at Unnan City Hospital.

Study period

The study covered a period from January 2021 to April 2024.

Setting

In 2022, the total population of Unnan City was 35,738 (17,231 males and 18,507 females), and the percentage of residents aged 65 years and older was 40.27%. There was only one public hospital in the rural area. The rural hospital had 281 beds during the study period, including 155 acute, 48 general, 30 rehabilitation, and 48 chronic patients. The Department of Family Medicine manages internal medicine patients through collaborative efforts with multiple healthcare professionals [9].

Study population

The study included patients aged 65 to 104 years admitted to Unnan City Hospital with a diagnosis of pyelonephritis during the study period. The inclusion criteria consisted of patients admitted for acute pyelonephritis and the availability of complete medical records. The exclusion criteria included patients with incomplete data, those admitted for other primary diagnoses, and those using medicines for diabetes.

Measured variables

Outcomes

The primary outcome was the incidence of hypoglycemic events during hospitalization, defined as a blood glucose level below 70 mg/dL.

Comorbid Conditions

Data on comorbidities, including asthma, stroke, cancer, chronic obstructive pulmonary disease (COPD), dementia, diabetes mellitus, and heart disease, were collected from the electrical medical records of Unnan City Hospital [10]. Asthma was identified based on patient history and spirometry results, while stroke included both ischemic and hemorrhagic events confirmed by neuroimaging. Cancer encompassed all malignancies, and COPD was diagnosed through clinical history and spirometry. Dementia was documented if there were significant cognitive impairments affecting daily functioning. Diabetes was confirmed by medication use or elevated fasting blood glucose/HbA1c levels, and heart disease included ischemic heart disease, heart failure, and arrhythmias.

Demographic and Clinical Characteristics

Demographic data included age, sex, and body mass index (BMI). Blood glucose levels were monitored throughout hospitalization, with baseline and lowest values documented to assess glycemic control.

Statistical analysis

Fisher’s exact test was used for categorical variables. For continuous variables, parametric data were analyzed using the Welch t-test, and nonparametric data were analyzed using the Mann-Whitney U-test. Multivariate logistic regression analysis was utilized to identify independent risk factors associated with hypoglycemic events among the study population. Variables for the model were chosen based on their clinical relevance and statistical significance in the preliminary univariate analysis, with a cutoff p-value of less than 0.05. The selected variables included age, sex, BMI, and comorbid conditions such as diabetes, heart disease, and COPD. The strength of association between each variable and the occurrence of hypoglycemia was quantified using odds ratios (OR) along with 95% confidence intervals (CI). A p-value of less than 0.05 was considered statistically significant for all analyses. Statistical analyses were performed using Easy R software, ensuring a robust and reliable evaluation of potential risk factors influencing hypoglycemic events in elderly patients [11].

Ethical considerations

This study was approved by the Unnan City Hospital Ethics Committee (approval number: 20240013). Due to its retrospective nature, informed consent was waived. All patient data were anonymized before analysis to protect patient confidentiality and privacy.

## Results

Demographic data

A total of 294 elderly patients diagnosed with acute pyelonephritis were included in the study. Among them, 146 patients (49.7%) experienced hypoglycemic events during their hospitalization, while 148 patients (50.3%) did not. The mean age in the hypoglycemic group was slightly lower (84.68 ± 9.30 years) compared to the non-hypoglycemic group (86.59 ± 9.17 years), but this difference did not reach statistical significance (p = 0.076). Gender distribution was similar across both groups, with 72 males (48.6%) in the non-hypoglycemic group and 82 males (56.2%) in the hypoglycemic group (p = 0.202). The mean BMI of patients in the hypoglycemic group was significantly lower (18.12 ± 3.29) than that of patients in the non-hypoglycemic group (20.00 ± 3.81), with a p-value of <0.001. Regarding comorbidities, asthma was present in 3 patients (2.0%) in the non-hypoglycemic group and 1 patient (0.7%) in the hypoglycemic group (p = 0.622). Stroke occurred in 28 patients (18.9%) in the non-hypoglycemic group and 19 patients (13.0%) in the hypoglycemic group (p = 0.203). Cancer was present in 4 patients (2.7%) in the non-hypoglycemic group and 6 patients (4.1%) in the hypoglycemic group (p = 0.540). COPD was observed in 2 patients (1.4%) in each group (p = 1.000). The in-hospital mortality rate was significantly higher in the hypoglycemic group, with 68 patients (46.6%) compared to 46 patients (31.1%) in the non-hypoglycemic group (p = 0.008) (Table 1).

**Table 1:** The demographics of the participants Note: BMI, body mass index; COPD, chronic obstructive pulmonary diseases, SD standard deviation. Data are presented as N (%) or Mean (±SD). P-values were calculated using t-tests for continuous variables and chi-square tests for categorical variables. Statistical significance was considered for p-values less than 0.05.

Factor	Non-hypoglycemic	Hypoglycemic	Test Statistic	P value
N	148	146		
Male sex (%)	72 (48.6)	82 (56.2)	χ² = 1.62	0.202
Age, mean (SD)	86.59 (9.17)	84.68 (9.30)	t = 1.78	0.076
BMI, mean (SD)	20.00 (3.81)	18.12 (3.29)	t = 4.76	<0.001
Glucose, mean (SD)	145.37 (52.52)	46.15 (13.51)	t = 20.55	<0.001
Asthma (%)	3 (2.0)	1 (0.7)	χ² = 0.24	0.622
Brain stroke (%)	28 (18.9)	19 (13.0)	χ² = 1.62	0.203
Cancer (%)	4 (2.7)	6 (4.1)	χ² = 0.37	0.54
COPD (%)	2 (1.4)	2 (1.4)	χ² = 0.00	1
Death (%)	46 (31.1)	68 (46.6)	χ² = 7.03	0.008
Dementia (%)	6 (4.1)	3 (2.1)	χ² = 0.45	0.501
Diabetes (%)	13 (8.8)	10 (6.8)	χ² = 0.19	0.665
Heart failure (%)	44 (29.7)	46 (31.5)	χ² = 0.06	0.801

Logistic regression analysis

Multivariate logistic regression analysis was performed to identify independent risk factors associated with hypoglycemia in the study population. After adjusting for potential confounders, lower BMI emerged as a significant independent risk factor for hypoglycemia. Specifically, for every unit decrease in BMI, the odds of experiencing hypoglycemia increased (OR = 0.84, 95% CI: 0.78-0.90, p < 0.00001), indicating an inverse relationship between BMI and hypoglycemia risk. Age also showed a significant, albeit modest, protective effect against hypoglycemia (OR = 0.97, 95% CI: 0.94-0.99, p = 0.015), suggesting that younger elderly patients within the study cohort were more likely to experience hypoglycemic events than their older counterparts. Other variables, including sex, asthma, stroke, cancer, COPD, dementia, diabetes, and heart failure, did not show a statistically significant association with hypoglycemia in the logistic regression model (Table 2).

**Table 2:** The logistic regression model for hypoglycemic episodes and the related factors. Note: BMI, body mass index; CI, confidence interval; COPD, chronic obstructive pulmonary diseases. P-values were calculated using z-tests from the logistic regression model. Statistical significance was considered for p-values less than 0.05.

Factor	Odds ratio	95%CI	Test Statistic	P value
Age	0.97	0.94-0.99	z = -2.44	0.015
Male Sex	1.26	0.76-2.08	z = 0.90	0.37
BMI	0.84	0.78-0.90	z = -4.42	0.0000053
Asthma	0.19	0.01-2.43	z = -1.28	0.2
Brain stroke	0.65	0.33-1.30	z = -1.20	0.23
Cancer	1.92	0.42-8.81	z = 0.84	0.4
COPD	1.40	0.13-14.90	z = 0.28	0.78
Dementia	0.47	0.10-2.14	z = -0.97	0.33
Diabetes	0.67	0.26-1.74	z = -0.83	0.41
Heart failure	1.41	0.82-2.42	z = 1.23	0.22

## Discussion

The present study aimed to identify the risk factors associated with hypoglycemia in elderly patients hospitalized with acute pyelonephritis. Our findings indicate that a lower body mass index (BMI) is significantly associated with an increased risk of hypoglycemia in this population. This is consistent with previous studies that have highlighted the vulnerability of elderly patients, particularly those with low BMI, to hypoglycemic events during hospitalization [12,13]. The association between lower BMI and hypoglycemia can be attributed to several factors, including decreased muscle mass, reduced hepatic glycogen stores, and altered pharmacokinetics of glucose-lowering medications in underweight individuals [14]. These physiological changes, coupled with the stress of acute illness, can exacerbate the risk of hypoglycemia in elderly patients.

Interestingly, while age itself did not show a significant impact on hypoglycemia risk, the inverse relationship observed in our logistic regression analysis suggests that younger elderly patients may be at a slightly higher risk than their older counterparts. This could be due to the possible presence of more aggressive medical management strategies or a higher likelihood of polypharmacy in relatively younger elderly patients [15]. Previous research has suggested that intensive glycemic control in elderly patients with multiple comorbidities can lead to increased hypoglycemic episodes without significant benefits in reducing complications [16]. Therefore, general physicians should consider a more individualized approach to glycemic management in this population [17].

Our study did not find a significant association between hypoglycemia and other comorbidities such as diabetes, heart failure, and chronic obstructive pulmonary disease (COPD). This contrasts with earlier studies that identified diabetes and comorbid conditions as primary contributors to hypoglycemic events in hospitalized patients [18,19]. One possible explanation for this discrepancy could be the difference in the study population and the specific focus on patients with pyelonephritis without diabetic medicines in our study. Furthermore, the stringent inclusion criteria and comprehensive control of confounding variables in our multivariate analysis might have mitigated the apparent effects of these comorbidities.

A notable finding of our study is the significantly higher in-hospital mortality rate associated with hypoglycemia. This result aligns with previous literature indicating that hypoglycemia is a strong predictor of poor outcomes in hospitalized patients [20]. Hypoglycemia can precipitate a cascade of adverse events, including cardiovascular complications, neurological damage, and increased susceptibility to infections, all of which contribute to higher mortality rates [20]. These findings underscore the need for vigilant monitoring and prompt management of hypoglycemia to prevent its severe complications, especially in the elderly. Blood glucose management should be monitored through interprofessional collaboration. Especially in rural contexts, a lack of medical professionals demands various forms of collaboration and role-sharing for effective care of older patients.[21,22]. General physicians in rural contexts should collaborate with multiple medical professionals for glucose management [23,24].

This study has several limitations. First, the retrospective design inherently limits the ability to establish causality between the identified risk factors and hypoglycemia. The reliance on medical records may also introduce data completeness and accuracy biases, particularly regarding comorbid conditions and medication use. Moreover, our study was conducted at a single hospital, which may limit the generalizability of the findings to other settings, particularly those with different patient demographics and clinical practices. Another limitation is the lack of information on nutritional status and dietary intake, which could have significantly impacted the risk of hypoglycemia. Previous studies have shown that malnutrition and inadequate caloric intake are critical contributors to hypoglycemic events in hospitalized patients [25]. Additionally, the study did not account for the types and doses of medications, particularly glucose-lowering drugs, which could have influenced the outcomes. Future research should aim to include these variables to provide a more comprehensive understanding of the risk factors associated with hypoglycemia in elderly inpatients. Lastly, while we identified low BMI as a significant risk factor, the study did not explore the potential mechanisms underlying this association. Further research, possibly involving biomarkers of nutritional status and muscle mass assessment, would be beneficial in elucidating the pathophysiological pathways linking low BMI to hypoglycemia in elderly patients.

## Conclusions

Our study highlights that low BMI is a significant independent risk factor for hypoglycemia in elderly patients hospitalized with acute pyelonephritis. This finding suggests that underweight elderly patients require careful monitoring and possibly tailored therapeutic interventions to prevent hypoglycemic episodes during hospitalization. The high in-hospital mortality associated with hypoglycemia underscores the urgent need for improved management strategies to mitigate its impact on this vulnerable population. Our findings emphasize the importance of a multidisciplinary approach in managing elderly inpatients, involving tight glucose monitoring, nutritional support, and medication review. Future studies should focus on prospective, multicenter designs to validate these findings and explore potential preventive measures that can be implemented in routine clinical practice. By addressing the risk factors identified in this study, healthcare providers can enhance patient safety and outcomes, ultimately improving the quality of care for elderly patients.
